# Mobile Genome Express (MGE): A comprehensive automatic genetic analyses pipeline with a mobile device

**DOI:** 10.1371/journal.pone.0174696

**Published:** 2017-04-12

**Authors:** Jun-Hee Yoon, Thomas W. Kim, Pedro Mendez, David M. Jablons, Il-Jin Kim

**Affiliations:** 1 Thoracic Oncology Laboratory, Department of Surgery, University of California San Francisco, San Francisco, California, United States of America; 2 Comprehensive Cancer Center, University of California San Francisco, San Francisco, California, United States of America; 3 CureSeq Inc., Brisbane, California, United States of America; Lawrence Berkeley National Laboratory, University of California, Berkeley, UNITED STATES

## Abstract

The development of next-generation sequencing (NGS) technology allows to sequence whole exomes or genome. However, data analysis is still the biggest bottleneck for its wide implementation. Most laboratories still depend on manual procedures for data handling and analyses, which translates into a delay and decreased efficiency in the delivery of NGS results to doctors and patients. Thus, there is high demand for developing an automatic and an easy-to-use NGS data analyses system. We developed comprehensive, automatic genetic analyses controller named Mobile Genome Express (MGE) that works in smartphones or other mobile devices. MGE can handle all the steps for genetic analyses, such as: sample information submission, sequencing run quality check from the sequencer, secured data transfer and results review. We sequenced an Actrometrix control DNA containing multiple proven human mutations using a targeted sequencing panel, and the whole analysis was managed by MGE, and its data reviewing program called ELECTRO. All steps were processed automatically except for the final sequencing review procedure with ELECTRO to confirm mutations. The data analysis process was completed within several hours. We confirmed the mutations that we have identified were consistent with our previous results obtained by using multi-step, manual pipelines.

## Introduction

There were several breakthroughs for genetic and genomic analyses since the first description of DNA double helix structure in 1953 [1,2]. A development of the polymerase chain reaction (PCR) technology opened an era for different types of genetic analyses with limited amount of DNA from various biological samples [3–5]. PCR amplification technology is still a foundation of up-to-date genome analysis technologies. Sanger sequencing was then developed [6,7], and enabled a discovery of human genome sequences and completed the human genome sequence map (The Human Genome Project) in 2003 (https://www.genome.gov/10001772). The next revolution of genetic and genomic analyses would be the development of microarray or DNA chip technology [8]. With this hybridization-based high throughput expression or genotype technologies, all known genes could be analyzed at one time for their expression. Microarray technology contributed to the identification of new subtypes and biomarkers of diseases such as cancer [8–12].

Next-generation sequencing (NGS) is the state-of-the-art technology widely used for genetic and genome analyses in many life science fields. NGS was initially developed in mid-2000’s [13–15], and aimed to sequence the genome in one experiment. Later, the same technology was adapted to allow the sequencing of the whole transcriptome, also called RNA-seq [16–19] and the epigenome [20]. The previous technologies mentioned above also had their own ‘paradigm-shifting’ and ‘revolutionary’ points. But, NGS is the first technology enabling us to sequence the whole exome or genome in one experiment [13–15]. Despite technical superiority by NGS, it was not easy to use NGS technology in a regular laboratory at the beginning for several reasons. First, it was strictly too complex. It required multiple challenging steps and relatively higher quality and quantity of DNA or RNA. Second, the cost was tremendously high. Although NGS can generate great amount of genome or exome data, the costs for reagents, chips or sequencing cells, and machines were extremely high, which prevented a frequent and regular use of NGS for various applications. Lastly, NGS data analyses or bioinformatics procedures were notoriously difficult for most biologists. As one whole exome or genome sequencing can produce up to gigabytes of data, it is overwhelming to handle by most molecular biologists or geneticists.

However, situations for NGS applications had changed dramatically for last several years. The sequencing cost has been significantly reduced from more than several thousands of dollars per exome to near one thousand dollars [21]. The size of sequencing data has also been dramatically reduced because of the increasing demand of a targeted sequencing focusing on 5–300 genes rather than exome sequencing [22–25]. Exome sequencing strategy is still invaluable for identifying new biomarkers by big research consortiums such as TCGA [26,27]. However, a targeted sequencing approach focusing only selected genes such as cancer panels or hereditary disease panels seems more dominant for precision or personalized medicine applications [22–25]. In fact, a lot of Clinical Laboratory Improvement Amendments (CLIA) labs provide a targeted sequencing panel rather than an exome or whole genome sequencing due to data accuracy, cost, and turn-around-time (TAT) [22–25]. Thus, a reduction of sequencing cost, data size, TAT, and an increase of competition among many commercial companies and CLIA labs would expedite the processes to the era of having personal sequencing information. Currently, only family members under screening programs of hereditary cancer or some already diagnosed cancer patients are genetically tested by NGS with appropriate biological samples (i.e. blood or tumor). Nevertheless, we believe that more and more people will need or want to know their genetic profiles in a near future. A successful application of circulating tumor DNA sequencing for the early detection of cancer or prognosis surveillance [28–30], supports the idea that sequencing information will be available to people who want to know it. Though, the cyber-security must be improved and there should be clear legal regulations and consensus for handling, analyzing, interpreting, and delivering the genome information as it is very massive and delicate personal information.

Although it seems very promising to see the era for a regular NGS sequencing screening testing (like X-ray), in a regular clinical laboratory, there are some significant hurdles to overcome. Those are NGS data analyses, interpretation, and reporting. There should be a robust automatic data processor from the sequencer through bioinformatics data analysis pipelines, clinical interpretation, and genetic information delivery. Many CLIA labs use different bioinformatics pipelines to analyze NGS data [22–25,31–33]. And yet, each step tends to be separated and controlled manually, possibly by different persons. After a laboratory technician complete the sequencing, the data quality may be checked first and then downloaded to the bioinformatics pipelines. Then Bioinformatics team starts converting the raw sequencing data into their appropriate analysis programs going through several steps such as sequence alignment, filtering, variant calling, annotation, and the final review. The selected genetic variants will be passed to the interpretation or analytics team for making a report and providing clinical or biological meanings. The finalized genetic report will then be delivered to patients or doctors via mail, e-mail, or fax. All these multiple and complex steps would be feasible only for analyzing a small scale of samples. However, if we want to handle a large number of samples accurately and robustly, this kind of currently used bioinformatics methods will be a problem.

Thus, we decide to develop an automatic NGS data analysis program which works in a mobile device (i.e. cellular phone) that controls the whole process, from the sequencing run in a sequencer through NGS data analyses to the genetic information delivery. One of challenging points for making the whole automatic process from the ‘wet’ laboratory sequencing works to the ‘dry’ bioinformatics works may be an efficient communication between wet and dry laboratories. We aim to make a simple but very useful program working in a mobile device to check and control all the processes of NGS. Though it is still preliminary to deliver the genome information directly to patient or individual due to legal issues, it may be eventually possible to get their genome information through a simple and easy way like mobile phone, not though on-site hospital visit or a regular mail delivery. Thus, it will be required to develop a comprehensive system delivering personal genome information by satisfying and keeping the rule and regulation by authorized organizations.

In this work, we present an automatic NGS data analysis processor, MGE, to show that full steps for the automatic NGS data analyses are available through a simple pipeline with mobile application and server application.

## Materials and methods

### An overview of the Mobile Genome Express (MGE)

**Fig 1:**
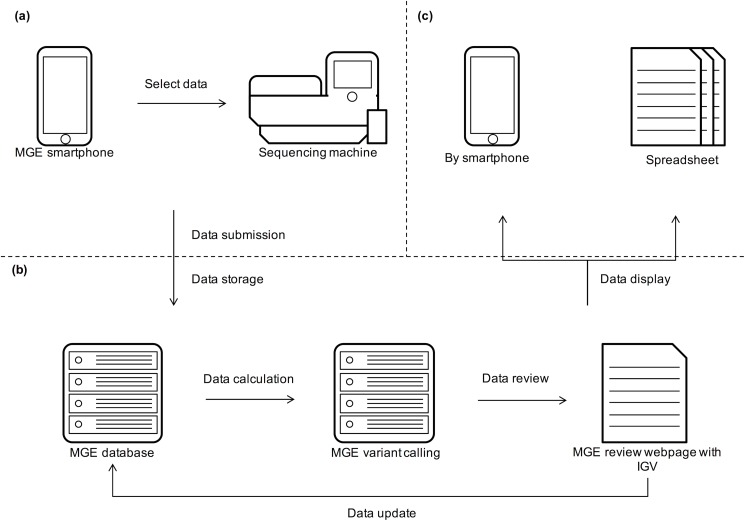
Workflow of MGE from sequencer to final result delivery. (a) Users access to the Sequencing instrument (e.g. Ion Torrent PGM) by using MGE smartphone application, to select a sequencing run/s to analyze. Then users’ server accesses to the Sequencer and downloads the corresponding data as selected on the smartphone. (b) Automatically, the Sequencing machine sends the data to MGE database. Then, the MGE variant-calling tool runs. Next, the variant calling output data is ready for user can check the sample result with MGE review page, including the sample’s status and comments. After the reviewer curation, result is submitted. (c) Users can download the result in spreadsheet file from users’ server to their smartphone.

The MGE system is composed with 5 steps of 1) data submission, 2) data calculation, 3) data storage, 4) data review, and 5) data display (Fig 1). These steps are organized as one pipeline called the MGE, with full automatic running system. We have developed the MGE process by using several computer languages. The smartphone application is made with Javascript and HTML5 by using Phonegap, the server side codes are made with Python and PHP5, and MGE uses MySQL for the database. Our testing server is operated by Ubuntu 14.04.

### Data submission

MGE is executed in a mobile device, connected to a main server (controlled by the user) and to the sequencer. To ensure the security of the data and access of the user, an authentication system has been implemented. After logging in, MGE requires to input the internal internet protocol (IP) address of the corresponding sequencer. Once the user access the sequencer, the sequencing run to be analyzed is selectable from the run list. The user can also check some of the sequencing run features (e.g. the number of samples included in the sequencing run and their barcode number).

**Fig 2:**
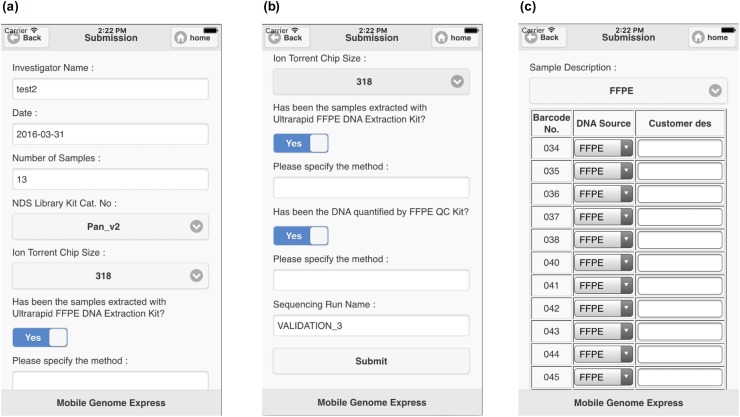
Sample submission form for analyzing sequencing data in MGE. After the user access the sequencer with MGE smartphone application and selects the samples to be analyzed, the app will guide the user through to verify the data imported into the submission form. The data can be modified into MGE database if needed. (a) First page of the submission form. Because the users use their own panel and chip size, there are sections where they can fill-in the information about the samples, which gets inserted into MGE database. (b) The second page of the submission form is managed to get detailed experiment information such as DNA extraction method as well as DNA quantity and quality control method used (Nanodrop, Picogreen or qPCR). (c) Third page. The sample type information can also be recorded for further analysis and evaluation of sequencing data quality.

The integration of some of the supported Application Program Interface (API) available in the Software Development Kit (SDK) of Ion Torrent Suite (Thermo Fisher Scientific Inc.) in MGE was coded by using JavaScript Object Notation (JSON) format. Therefore, MGE smartphone application provides user interaction, and setting a target to go to next steps for user’s main server. Furthermore, we designed a sample submission form to gather experiment information in this step (Fig 2). Most information is obtained from the sequencing platform automatically; although, the user can instead introduce the sequencing information manually through the smartphone application. Once the information is submitted, MGE transfers data from sequencer’s server to the MGE’s main server, followed by running it in an authorized path.

### Data calculation

Data calculation can be customized with different plugins such as: sequencing depth calculator, low quality sequencing reads filter, variant caller, and many more. MGE manage the server’s calculation process by running PHP5 and Python codes, and can be applied in with samples automatically, since MGE passes to calculating codes file paths and essential information. After getting results in this step, server inserts data to MGE database. MGE sends notification to MGE administrator by email each time when the data calculation starts and when it ends. The recipient list of the MGE alert email, can include as many users as needed, under administrator supervision.

### Data review

**Fig 3:**
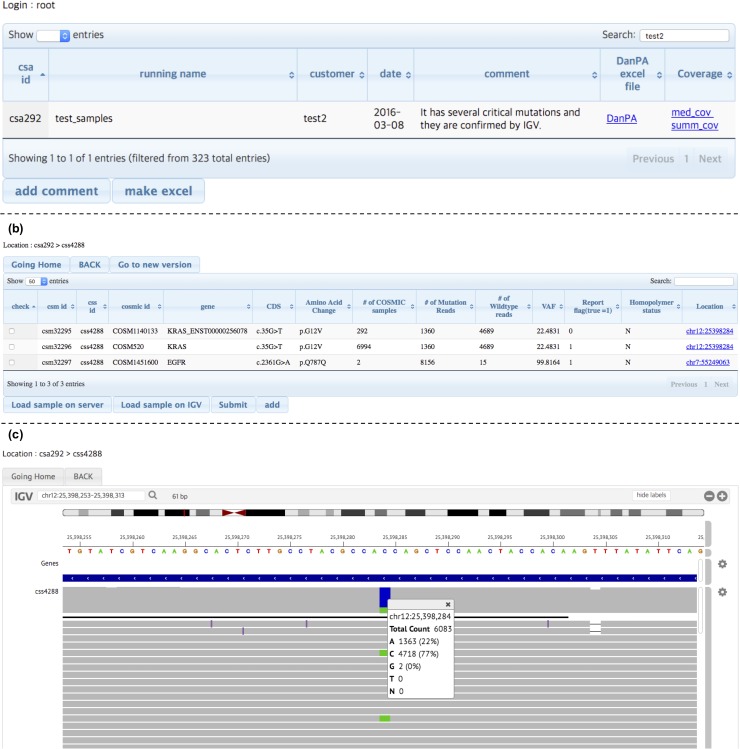
Review of sequencing data in ELECTRO (MGE). (a) ELECTRO’s web interface, the MGE original sequencing data review web page. (b) Variant curation page. Important information is displayed per each variant. MGE reflects this information on final result screen on user’s smartphone and spreadsheet file. (c) Review of raw sequencing data on IGV plugin within the ELECTRO-MGE interface. Csm id: MGE own ID for a mutation; css id: MGE own ID for a sample; cosmic id: COSMIC ID; gene: Gene symbol (HGVS); CDS: CDS nomenclature of each variant; Amino Acid Change: amino acidic nomenclature of each variant; # of COSMIC samples: number of positive records of the variant in COSMIC database; # of mutation Reads: number of sequencing reads with variant allele; # of Wildtype reads: number of sequencing reads with reference allele; VAF: variant allele frequency; Report flag (true = 1): Present decision of a mutation, 1 = true mutation, 0 = false mutation; Homopolymer status: region within a homopolymer region (Y = yes, N = no); Location: genomic coordinates (chr#:start_nt) of the variant.

MGE has a separate web-based application to review the sample-related output sequencing data, called ELECTRO (Fig 3). The application is designed in a table-like interface, where the users can easily browse the data by user’s sequencing name, submission date, and user ID. All the tabs are sortable, which makes the data reviewing process more efficient and convenient. In addition, ELECTRO integrates an Integrative Genomic Viewer (IGV) interface, that allows the review of each called mutation, by clicking the IGV button. Once the review is finished, ELECTRO incorporates the results editor and generator, which exports the final mutation data to an independent spreadsheet file. With these features users can edit sample’s mutation status details and export the final result. ELECTRO allows the simultaneous online connection of all the data-review team members. This feature allows a real-time review of any sequencing result (Fig 3).

### Data storage

MGE has its own database and automatically stores all the data into it, such as: sequencing file, information from sequencing data, mutation result, and final result.

Data can be stored at any step, even if the calculation is being performed. Upon data submission and calculation, MGE inserts the information to its database and user can access to this database by ELECTRO. We built this database scheme to optimize all MGE procedure in practice.

### Data distribution and security

**Fig 4:**
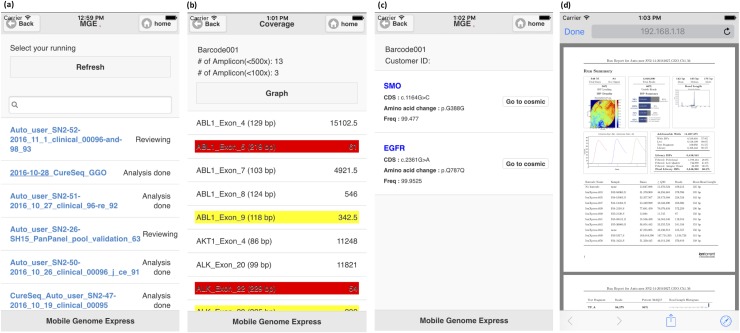
Example of sequencing results output from MGE. MGE shows the sample results on its smartphone application. Users can check (a) comments from bioinformatics analyst, (b-c) mutation and coverage reports of the sample, and (d) quality matric PDF from PGM server. All these reports get generated automatically by a smartphone and MGE server. Users can also check the status of their samples analyzing progress on a smartphone. The server will update the messages depending on the status of the progress, such as: “Running”, “Coverage completed”, “Reviewing”, and “Analysis done”.

Data distribution, the final step of MGE process, is similar to ELECTRO. The only difference is that it runs in a smartphone. MGE smartphone application can show final reviewed data such as: Catalogue of Somatic Mutations in Cancer (COSMIC) mutation ID, amino acid change and mutation’s allele frequency (Fig 4). Every information gets processed and reviewed by the bioinformatics and data reviewing specialists, and the MGE results include direct comments from the data curation team. This information can be displayed both in the smartphone and in the output spreadsheet, which is very important for a proper contextualization of each mutation and variant annotation, when needed. Moreover, read-depth report for each amplicons and quality PDF from sequencing machine are provided to users for checking sample’s quality. For information security, we used Virtual Private Network (VPN) to secure our all information and MGE can be install in VPN environment.

### Targeted cancer panel sequencing

The Acrometrix Oncology Hotspot Control DNA (Thermo Fisher, Waltham, MA; Cat. No. 969056) was used for generating sequencing data for testing and evaluating MGE and ELECTRO as previously described [22]. This control DNA contains more than 500 COSMIC mutations from 53 genes (Thermo Fisher, Waltham, MA; Cat. No. 969056). A targeted cancer panel with selected 25 genes (NextDay Seq-Pan Cancer HotSpot Panel kit, CureSeq Inc.) was used for a deep sequencing of the The Acrometrix Oncology Hotspot Control DNA as previously described [22,34]. In brief, 10 ng of control DNA was amplified and ligated with barcoded and adaptors with the library prep kit (NextDay Seq-Pan Cancer HotSpot Panel kit, CureSeq Inc.). The prepared library was cleaned up using magnetic beads and resuspended in 1x low-TE buffer, followed by qualitative and quantitative electrophoretic analysis by High Sensitivity DNA chip (Agilent Technologies, Santa Clara, CA; Cat. No. 5067–4626). After the emulsion PCR and library enrichment, the prepared library was sequenced using an Ion 314 v2 Chip (Thermo Fisher), and sequenced on an Ion Torrent™ PGM sequencer. The generated sequencing data was processed with MGE from data submission to data distribution as described above.

### Access to MGE and ELECTRO

MGE and ELECTRO are available for purchase at CureSeq Inc. (www.cureseq.com, Cat #: SKU#: CS-MGE-01). The register is allowed through the administrator and the administrator will create an account upon request. The account credentials will be given for the user to access the application. Also, the application is protected by virtual private network (VPN), and the address for VPN access will also be provided by administrator. MGE is a smartphone application so a mobile device is used for MGE, and ELECTRO can be used by PHP path installed in a personal computer. Additionally, the user ID and password for ELECTRO is embedded in the system.

## Results and discussion

### Actual data process with sequencing data

Once the sequencing reaction was completed, the MGE smartphone application notifies it by email. Next, MGE saves the data into its database, and execute DanPA (CureSeq Inc.), a variant caller specifically coded to call variants from targeted sequencing experiments, previously annotated in the COSMIC database [35]. MGE transfer the.bam and.bai files from the PGM sequencer server to the MGE’s main server and send an alert to the bioinformaticians. The mutation data was curated by using ELECTRO.

We used a 25-gene targeted cancer panel aiming to detect clinically relevant mutations [22]. Thus, we designed our variant detecting algorithm for detecting known and reported mutations rather than un-validated (both technically and clinically) variants. That is why we decided to focus on mutations registered in the COSMIC database, one of the most extensive and robust cancer-related, somatic mutation repository with approximately 3.5 million mutations reported and registered at COSMIC database. If users want to use other variant callers such as GATK or MuTect [31–33], MGE also supports them.

### Data review from submitted data

By checking the data under ELECTRO, bioinformaticians can review the sequencing results easily and make a comment for communicating with researchers, medical doctors, or other bioinformaticians.

First, although our variant caller algorithm filters out most of variants located within homopolymer regions (to avoid false positives in those regions, caused by the difficulties of the Ion Torrent PGM chemistry to resolve accurately the insertion/deletions (indels) at homopolymer regions), special attention was paid while reviewing such variants located in a tandem repeat or a homopolymer region [36]. Second, MGE database includes artificial intelligence (AI) features that allows the identification of false positive variants specific for a given sequencing panel. If during the variant call, MGE detects a match between a detected variant and any variant record in the mentioned database, a warning message is delivered by MGE into ELECTRO. The history of false positive variants could be optionally used as a filtering factor. As NGS tends to detect the same false positives variants in a recurrent fashion in the same genomic regions, a record of false positives especially at the homopolymer regions resulted extremely helpful to increase the accuracy of variant calling. Third, we also considered the number of times that a single mutation has been previously reported in the COSMIC database as a deciding factor of robust mutation call. The output format of the ELECTRO-MGE mutation file can be customized according to the user’s preferences. The whole analysis was completed within an hour after receiving the raw sequencing result data from the sequencer.

### Identified mutations

We used all the variants registered in COSMIC (version 76) database and filtered out germline polymorphisms or sequencing error or false positives in the final validation analyses. We checked all the detected variants using IGV to confirm the true positive results. If necessary, additional validation sequencing was done and analyzed to confirm the results. The library construction panel targeted 83 out of the 500 COSMIC mutations included in the Acrometrix control DNA (Table 1). All 83 mutations were detected, including 7 synonymous and 76 non-synonymous. Among the non-synonymous, 2 were insertions, 1 was a deletion, and 73 were point mutations. Additionally, 74 amplicons out of 75 amplicons in this sample had a sequencing depth of >500x. The results analyzed by MGE were 100% consistent with previous sequencing data of Acrometrix control DNA which was manually analyzed and reviewed [22].

**Table 1:** The identified mutations from the Acrometrix control DNA using MGE and Electro.

Gene	COSMIC ID	HGVS name (CDS)	Amino Acid Change
ABL1(NM_005157)	COSM12631	c.742C>G	p.L248V
ABL1(NM_005157)	COSM12577	c.749G>A	p.G250E
ABL1(NM_005157)	COSM12576	c.757T>C	p.Y253H
ABL1(NM_005157)	COSM12573	c.763G>A	p.E255K
ABL1(NM_005157)	COSM12602	c.827A>G	p.D276G
ABL1(NM_005157)	COSM235737	c.878_879insGCC	p.I293>MP
ABL1(NM_005157)	COSM49071	c.1150C>A	p.L384M
ABL1(NM_005157)	COSM12604	c.1187A>G	p.H396R
AKT1(NM_005163)	COSM33765	c.49G>A	p.E17K
ALK(NM_004304)	COSM28056	c.3824G>A	p.R1275Q
ALK(NM_004304)	COSM28055	c.3522C>A	p.F1174L
BRAF(NM_004333)	COSM476	c.1799T>A	p.V600E
BRAF(NM_004333)	COSM471	c.1790T>G	p.L597R
BRAF(NM_004333)	COSM467	c.1781A>G	p.D594G
BRAF(NM_004333)	COSM462	c.1742A>G	p.N581S
BRAF(NM_004333)	COSM450	c.1391G>T	p.G464V
BRAF(NM_004333)	COSM27986	c.1380A>G	p.G460G
BRAF(NM_004333)	COSM1448625	c.1359T>C	p.P453P
EGFR(NM_005228)	COSM13177	c.2063T>C	p.L688P
EGFR(NM_005228)	COSM41905	c.2092G>A	p.A698T
EGFR(NM_005228)	COSM6239	c.2156G>C	p.G719A
EGFR(NM_005228)	COSM13979	c.2170G>A	p.G724S
EGFR(NM_005228)	COSM28603	c.2293G>A	p.V765M
EGFR(NM_005228)	COSM1451600	c.2361G>A	p.Q787Q
EGFR(NM_005228)	COSM13190	c.2375T>C	p.L792P
ERBB2(NM_004448)	COSM20959	c.2324_2325ins12	p.A775_G776insYVMA
ERBB2(NM_004448)	COSM14065	c.2524G>A	p.V842I
ERBB2(NM_004448)	COSM686	c.2570A>G	p.N857S
FLT3(NM_004119)	COSM1166729	c.2516A>G	p.D839G
FLT3(NM_004119)	COSM783	c.2503G>T	p.D835Y
FLT3(NM_004119)	COSM19522	c.1775T>C	p.V592A
FLT3(NM_004119)	COSM25248	c.2492G>A	p.G831E
GNA11(NM_002067)	COSM21651	c.547C>T	p.R183C
GNA11(NM_002067)	COSM52969	c.626A>T	p.Q209L
GNAQ(NM_002072)	COSM52975	c.548G>A	p.R183Q
GNAQ(NM_002072)	COSM1463119	c.523A>T	p.T175S
HRAS(NM_005343)	COSM499	c.182A>G	p.Q61R
HRAS(NM_005343)	COSM495	c.175G>A	p.A59T
HRAS(NM_005343)	COSM249860	c.81T>C	p.H27H
HRAS(NM_005343)	COSM483	c.35G>T	p.G12V
IDH1(NM_005896)	COSM28746	c.395G>A	p.R132H
IDH1(NM_005896)	COSM1404902	c.388A>G	p.I130V
IDH1(NM_005896)	COSM96922	c.367G>A	p.G123R
IDH2(NM_002168)	COSM33733	c.515G>A	p.R172K
IDH2(NM_002168)	COSM41590	c.419G>A	p.R140Q
KRAS(NM_033360)	COSM19940	c.351A>C	p.K117N
KRAS(NM_033360)	COSM554	c.183A>C	p.Q61H
KRAS(NM_033360)	COSM546	c.175G>A	p.A59T
KRAS(NM_033360)	COSM1169214	c.101C>T	p.P34L
KRAS(NM_033360)	COSM14208	c.104C>T	p.T35I
KRAS(NM_033360)	COSM521	c.35G>A	p.G12D
KRAS(NM_033360)	COSM507	c.24A>G	p.V8V
MAP2K1(NM_002755)	COSM1235478	c.171G>T	p.K57N
MAP2K1(NM_002755)	COSM1678546	c.199G>A	p.D67N
NRAS(NM_002524)	COSM584	c.182A>G	p.Q61R
NRAS(NM_002524)	COSM1332933	c.174A>G	p.T58T
NRAS(NM_002524)	COSM577	c.52G>A	p.A18T
NRAS(NM_002524)	COSM564	c.35G>A	p.G12D
NRAS(NM_002524)	COSM24850	c.29G>A	p.G10E
PIK3CA(NM_006218)	COSM759	c.1616C>G	p.P539R
PIK3CA(NM_006218)	COSM760	c.1624G>A	p.E542K
PIK3CA(NM_006218)	COSM763	c.1633G>A	p.E545K
PIK3CA(NM_006218)	COSM1420865	c.1640A>G	p.E547G
PIK3CA(NM_006218)	COSM328026	c.3110A>G	p.E1037G
PIK3CA(NM_006218)	COSM775	c.3140A>G	p.H1047R
PTEN(NM_000314)	COSM5109	c.302T>C	p.I101T
PTEN(NM_000314)	COSM5266	c.314G>T	p.C105F
PTEN(NM_000314)	COSM5199	c.334C>G	p.L112V
PTEN(NM_000314)	COSM5123	c.395G>A	p.G132D
RET(NM_020975)	COSM1048	c.1894_1906>AGCT	p.E632_T636>SS
RET(NM_020975)	COSM1223553	c.1942G>A	p.V648I
RET(NM_020975)	COSM965	c.2753T>C	p.M918T
SMAD4(NM_005359)	COSM1389054	c.1001A>G	p.Q334R
SMAD4(NM_005359)	COSM1389057	c.1010A>G	p.E337G
SMAD4(NM_005359)	COSM14109	c.1018A>G	p.K340E
SMAD4(NM_005359)	COSM14111	c.1028C>G	p.S343*
SMAD4(NM_005359)	COSM14114	c.1504A>G	p.R502G
SMAD4(NM_005359)	COSM1389099	c.1519A>G	p.K507E
SMAD4(NM_005359)	COSM14134	c.1576G>T	p.E526*
SMAD4(NM_005359)	COSM1389106	c.1591C>A	p.R531R
SMO(NM_005631)	COSM13145	c.595C>T	p.R199W
SMO(NM_005631)	COSM216037	c.1234C>T	p.L412F
SMO(NM_005631)	COSM13146	c.1604G>T	p.W535L

The suspicious false positives variant calls were filtered out by ELECTRO according to the reviewing policy described above. In addition, MGE supports IGV launcher and searching mechanism, and it would boost the sharing of each decision making process of multiple users.

## Conclusions

It is highly expected that NGS sequencing information will be available to more and more people who want to know their genetic information, thanks to the significant reduction of a cost and TAT of NGS sequencing. We show that MGE can be an efficient mobile device or cell phone-based genetic controller enabling personalized genome analyses using targeted NGS panel feasible to most of regular laboratories. Current workflows in most laboratories require technicians or bioinformaticians to stay in a laboratory to check the sequencing run quality from the sequencing machine. Then data should be transferred to different types of bioinformatics pipelines used for each laboratory and analyzed by skilled bioinformaticians. All these manual-based sequencing quality check, data transfer, analyses, and review steps prevent the fast and robust genetic analyses. MGE allows us to check all the processes mentioned above into one application of a cellular phone or other mobile device. We used TMAP (Torrent Mapping Alignment Program) for mapping the raw sequencing reads and used DanPA program for variant calling and annotation. DanPA was previously tested in different sequencing projects [22, 37]. We plan to install and use another variant caller in MGE in a near future.

We believe that the automated MGE software, streamlines the genetic data flow from sequencer to clinical report, increasing robustness and efficiency. MGE is a very helpful tool that can help to increase the adoption of NGS technology in CLIA certified laboratories, as well as increase productivity of research, medical, and other life science sequencing laboratories.
